# Case Report: Cardiac angioma with multiple pulmonary angiomatous nodules: a rare presentation of systemic angiomatosis

**DOI:** 10.3389/fcvm.2026.1790569

**Published:** 2026-05-19

**Authors:** Tian Yang, Zheng Zhaowei, Wang Jun

**Affiliations:** Department of Thoracic and Cardiovascular Surgery, Hangzhou Hospital of Traditional Chinese Medicine, Hangzhou, China

**Keywords:** angioma, heart neoplasms, immunohistochemistry, lymphangioleiomyomatosis, pulmonary nodules

## Abstract

**Background:**

Primary cardiac tumors are clinically rare. When these tumors coexist with multiple bilateral pulmonary nodules, diagnosis becomes more complex and requires consideration of rare diseases, such as lymphangioleiomyomatosis and pulmonary epithelioid hemangioendothelioma.

**Case presentation:**

This report describes a 40-year-old female patient incidentally found to have a paraventricular cardiac mass and multiple solid lung nodules. A multidisciplinary team assessed her case. Because of the high risk of cardiac biopsy due to the mass's proximity to the coronary artery, a thoracoscopic wedge resection of a pulmonary nodule was performed instead. Immunohistochemical results—SMA(+), D2-40(focal +), CD31(+), CD34(+), Desmin(+), and HMB45(-)—confirmed angioma. Based on intraoperative findings and pulmonary pathology, the cardiac lesion was strongly suspected to be a homologous angioma. Thus, high-risk cardiac biopsy was avoided. The patient recovered well and remained stable, with no new lesions, over one year of follow-up.

**Conclusion:**

This case illustrates a rare co-occurrence of cardiac angioma and multiple pulmonary angiomas, expanding angioma's known systemic spectrum. It emphasizes the critical role of multidisciplinary collaboration in managing complex cardiopulmonary masses and the need for individualized, risk-averse surgical strategies. For patients with multiple pulmonary nodules and mediastinal or cardiac masses, angioma should be in the differential diagnosis. Detailed immunohistochemical analysis is crucial to accurately distinguish this tumor from other vascular and mesenchymal tumors.

## 1 Background

Angioma is a rare systemic disease that can occur in various tissues and organs throughout the body, including the liver, kidneys, skin, and subcutaneous tissues (1). However, cardiac involvement by angioma is exceedingly uncommon, accounting for less than 2% of all primary cardiac tumors (2). Cardiac angiomas typically present as solitary, well-defined mass lesions located in the cardiac chambers, myocardium, or pericardium. Their clinical manifestations are diverse and non-specific, ranging from incidental asymptomatic findings to symptoms related to mass effect, such as arrhythmias, heart failure, or even sudden death (3). Even more rare is the co-occurrence of a cardiac angioma with multiple bilateral pulmonary nodules, which poses a significant diagnostic challenge. This multicentric, multi-organ presentation necessitates a broadened differential diagnosis, requiring careful distinction from a range of other rare entities with similar imaging features. Lymphangioleiomyomatosis, which predominantly affects women of reproductive age and presents with multiple cystic or nodular lung lesions often accompanied by extra-pulmonary manifestations (e.g., renal angiomyolipomas), is an important condition to consider. Pulmonary epithelioid hemangioendothelioma is another rare, low-grade malignant vascular tumor that also manifests as multiple bilateral pulmonary nodules, sharing some biological features with angioma but differing in pathological origin. Furthermore, metastatic tumors, other primary cardiac tumors (such as myxoma or lipoma), and inflammatory granulomatous diseases must also be included in the differential evaluation.

Therefore, this article aims to provide a detailed report of a rare case of cardiac angioma coexisting with multiple bilateral pulmonary angiomatous nodules. By systematically describing the diagnostic and therapeutic process, particularly highlighting the pivotal role of individualized surgical decision-making and key immunohistochemical analysis in confirming the diagnosis, we hope to offer valuable insights for clinicians managing similar complex cases and to enhance the understanding of the systemic manifestations of angioma.

## 2 Case presentation

### 2.1 General information

**Figure 1:**
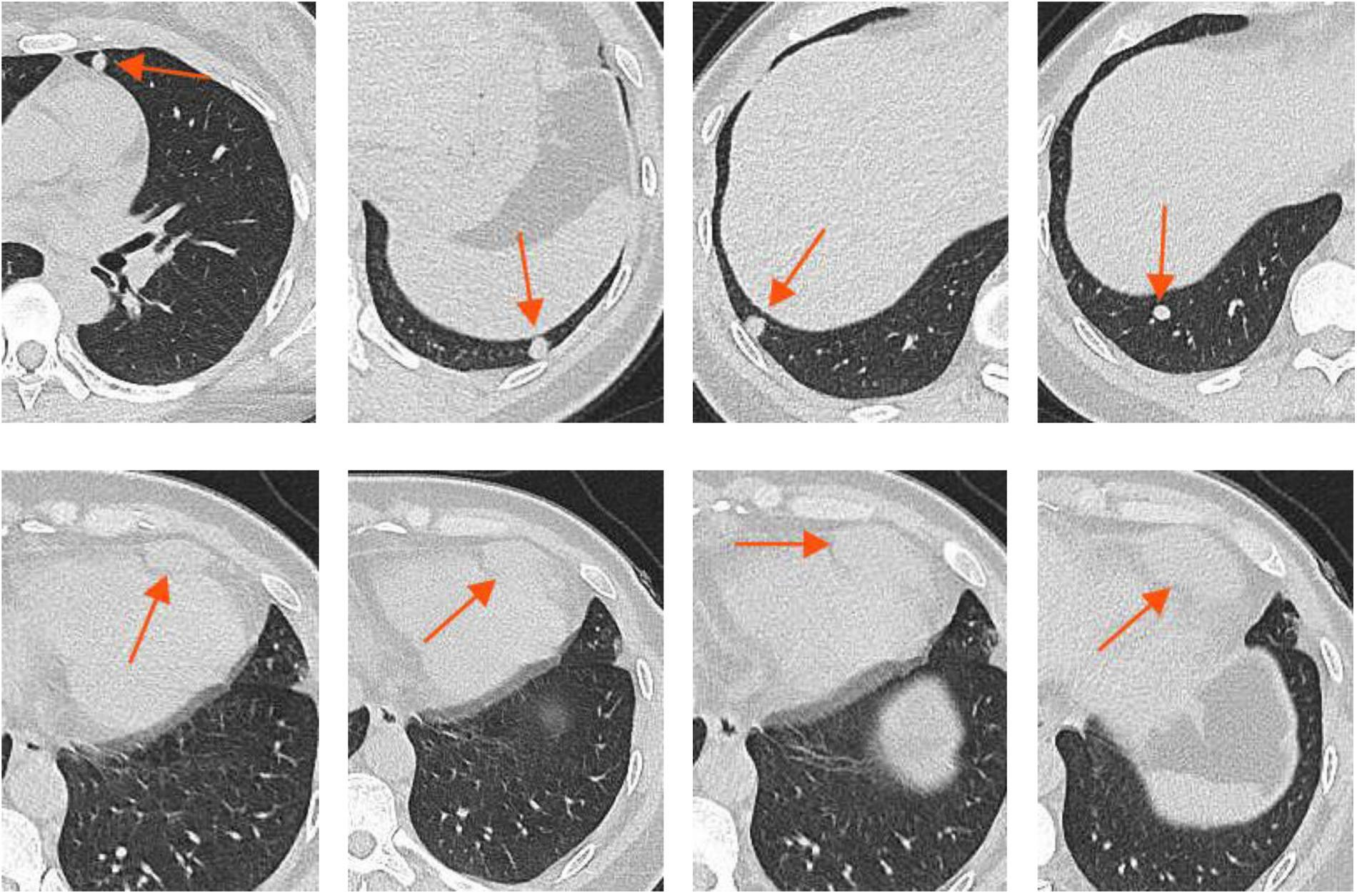
High-resolution non-contrast chest CT.

A 40-year-old female of Asian ethnicity was admitted due to an incidental finding of a paraventricular cardiac mass and multiple bilateral pulmonary nodules for one day. She reported no obvious chest tightness, shortness of breath, cough, or expectoration. Her past medical history was unremarkable, with no history of surgery, trauma, or allergies. Physical examination of the heart, lungs, and abdomen revealed no abnormalities, and no palpable lymphadenopathy was noted in the neck or supraclavicular fossae. Auxiliary examination: High-resolution Non-contrast Chest Computed Tomography(CT) showed a patchy soft-tissue density lesion anterior to the heart and multiple nodular foci in both lungs (Figure 1).

### 2.2 Preliminary diagnosis

1. Multiple bilateral pulmonary nodules; 2. Cardiac mass.

### 2.3 Admission investigations

#### 2.3.1 Tumor markers

Alpha-fetoprotein 2.00 ng/mL, Carcinoembryonic Antigen 1.54 ng/mL, Carbohydrate Antigen 125 10.70 U/mL, Carbohydrate Antigen 19-9 17.03 U/mL, Carbohydrate Antigen 15-3 6.16 U/mL, Ferritin 3.30 ng/mL, Carbohydrate Antigen 50 8.47 IU/mL, Cytokeratin 19 Fragment 0.83 ng/mL, Carbohydrate Antigen 242 9.59 IU/mL, Carbohydrate Antigen 72-4 1.14 IU/mL, Neuron-Specific Enolase 8.80 ng/mL, Squamous Cell Carcinoma Antigen 0.71 ng/mL.

#### 2.3.2 Contrast-enhanced chest CT

**Figure 2:**
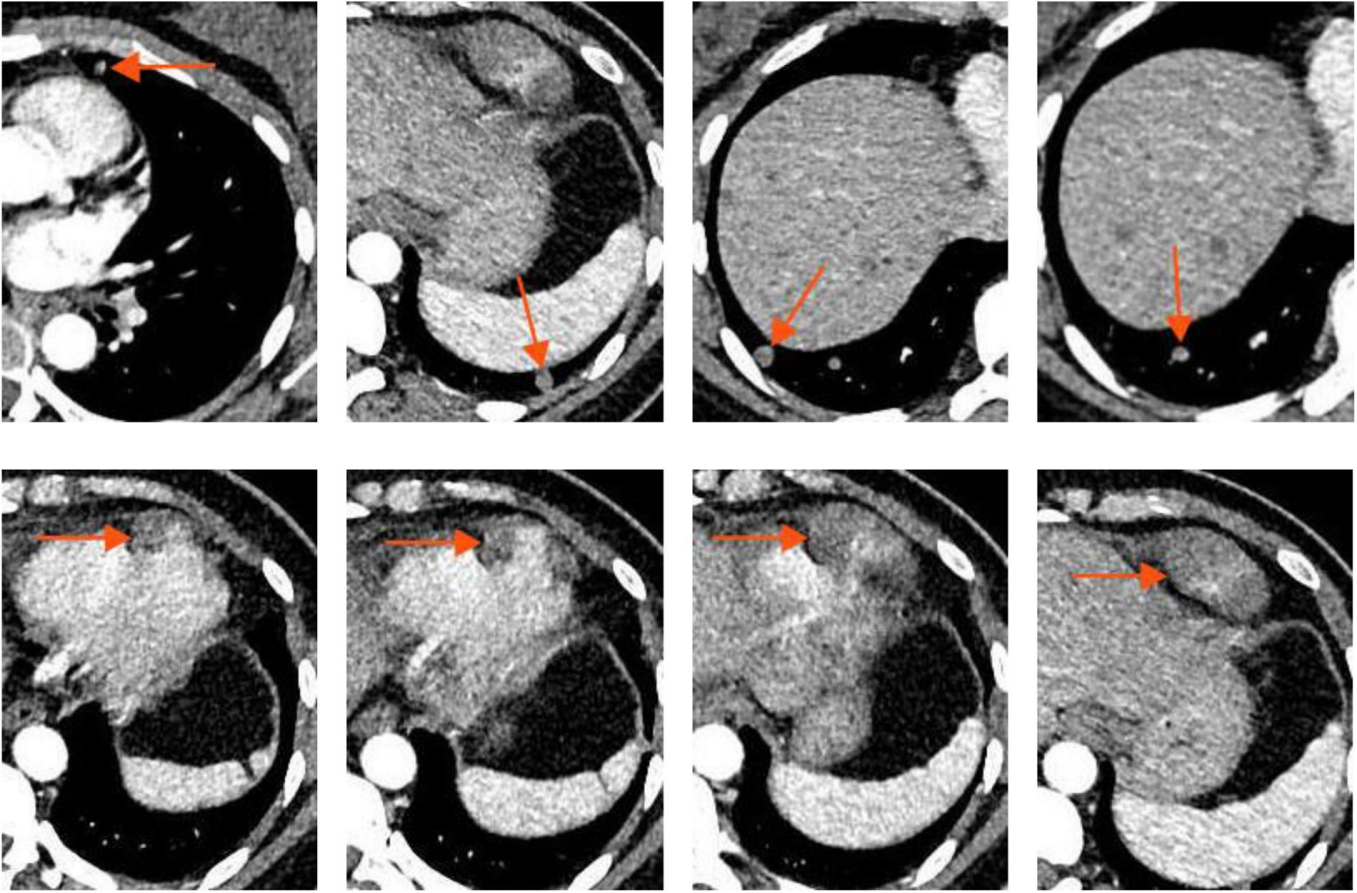
Contrast-enhanced chest CT.

A mass adjacent to the left ventricle with ill-defined borders from the left ventricular wall; Multiple small nodules were noted in both lungs (Figure 2).

#### 2.3.3 Mediastinal magnetic resonance imaging (MRI)

**Figure 3:**
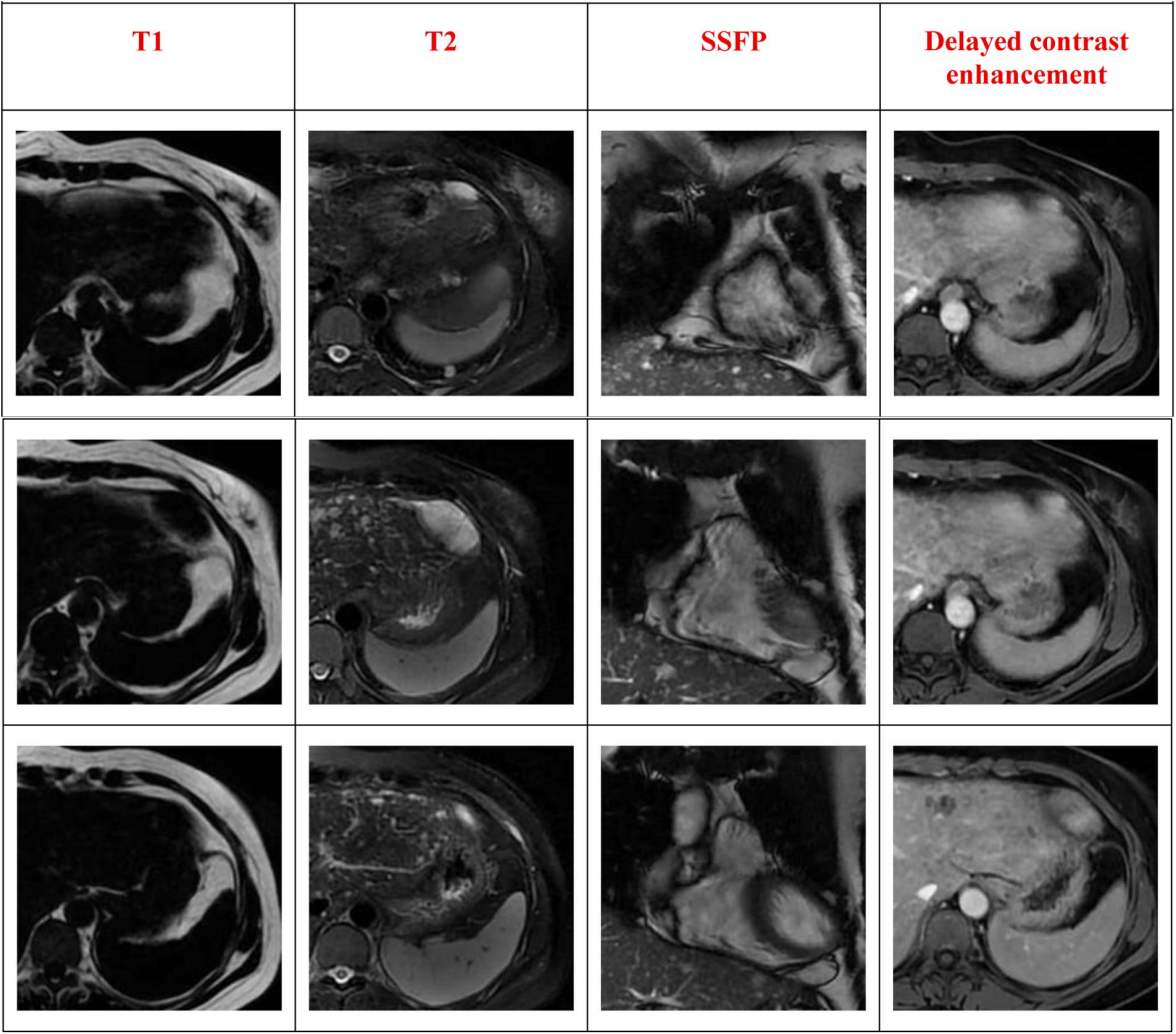
Mediastinal MRI.

A mass adjacent to the left ventricular apex, with indistinct borders from the left ventricle (Figure 3).

#### 2.3.4 Whole-body positron emission tomography/computed tomography (PET/CT) scan

**Figure 4:**
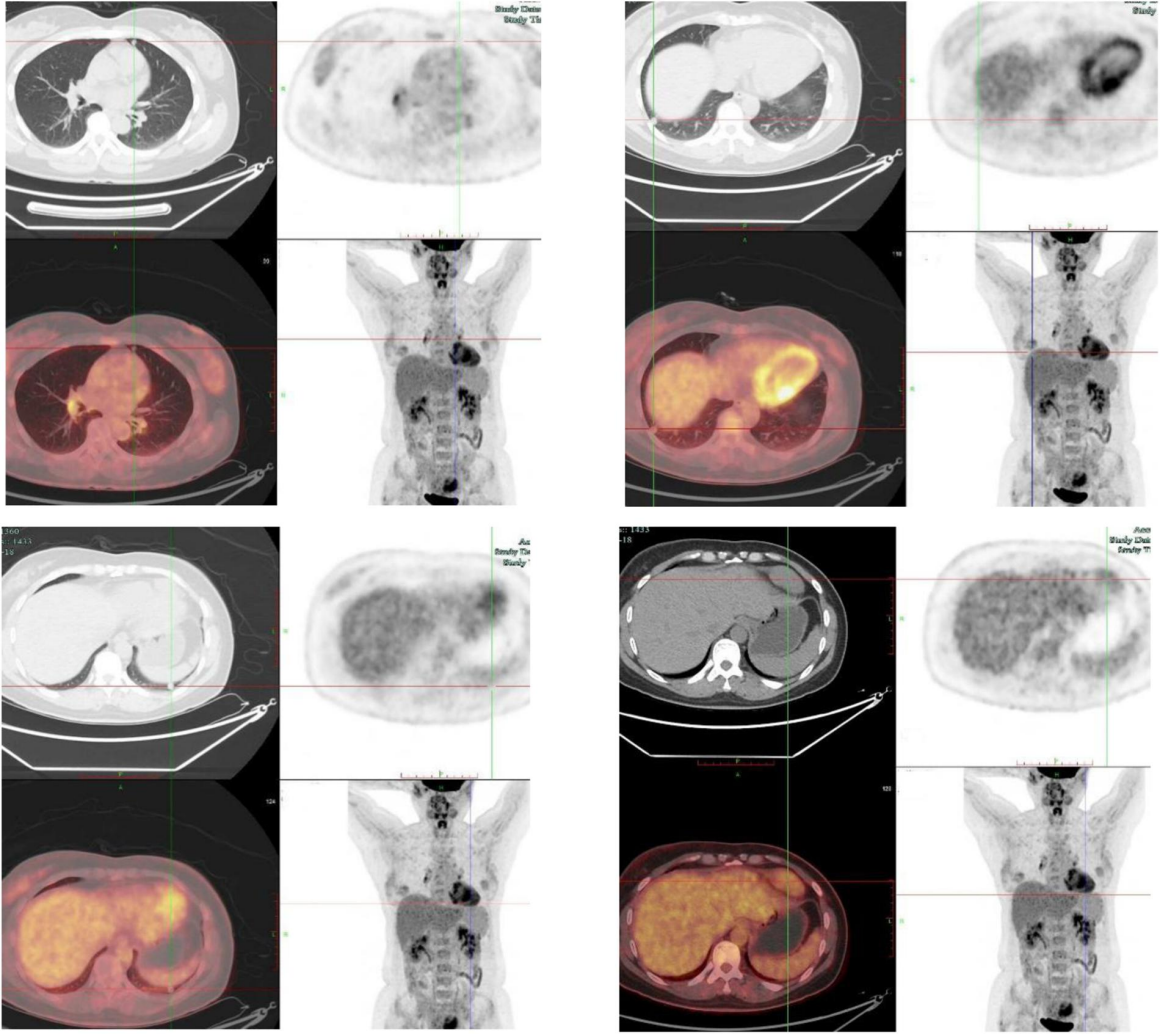
Whole-body PET/CT scan.

Multiple small nodules in both lungs, with no significant glucose metabolism, are suggestive of benign lesions. A mass at the left anterior border of the pericardium, showing increased glucose metabolism, is suggestive of a benign pericardial lesion (Figure 4).

#### 2.3.5 Coronary artery computed tomography angiography (CTA)

**Figure 5:**
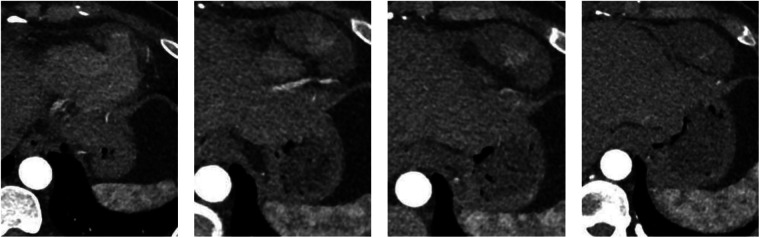
Coronary artery CTA.

A patchy soft tissue density shadow is observed adjacent to the left ventricle, through which the distal left anterior descending artery passes without significant luminal narrowing (Figure 5).

#### 2.3.6 Cardiac ultrasound

**Figure 6:**
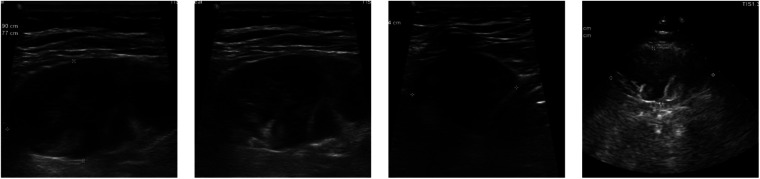
Cardiac ultrasound.

Mild tricuspid regurgitation and a space-occupying lesion adjacent to the cardiac apex within the pericardium (Figure 6).

### 2.4 Multidisciplinary consultation

**Figure 7:**
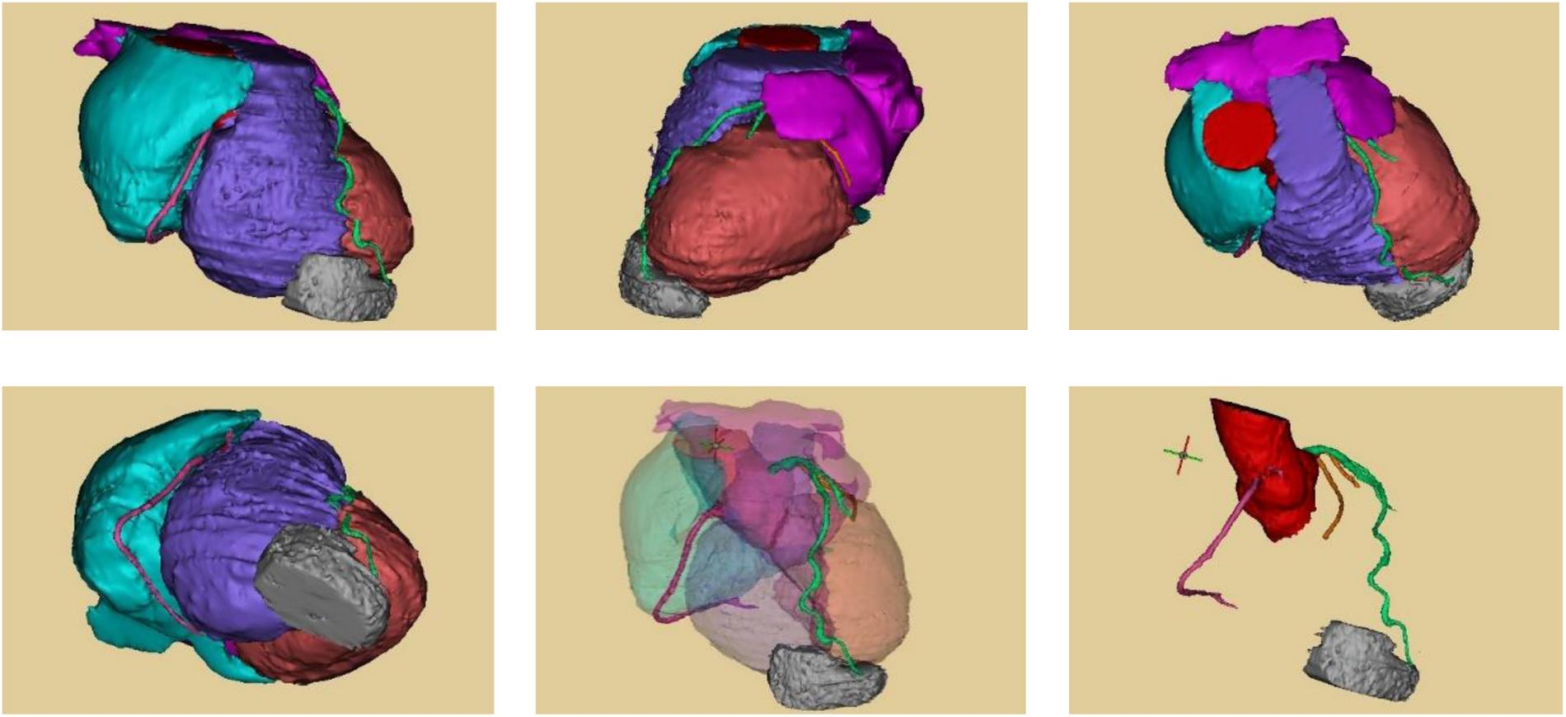
Three-dimensional reconstruction of the paraventricular mass.

The patient presented with multiple bilateral pulmonary nodules and a paraventricular cardiac mass. Imaging studies were unable to definitively characterize the nature of the nodules, making it difficult to differentiate between angioma, metastatic tumors, and primary pulmonary tumors. A multidisciplinary consultation involving our department, oncology, and radiology was conducted. It was unanimously agreed that although the pulmonary nodules were numerous, they were too small for reliable percutaneous biopsy, which would carry a high probability of false-negative results. Furthermore, preoperative biopsy of the paraventricular mass was deemed unfeasible. After discussion and obtaining the patient's consent, a decision was made to proceed with a scheduled exploratory thoracoscopy (Figure 7).

## 3 Surgical treatment

Surgical thoracoscopy was performed safely to obtain pulmonary tissue for pathological diagnosis. The procedure was uneventful, and histopathology confirmed a benign vascular lesion. No direct intervention on the cardiac mass was performed due to high procedural risk.

**Figure 8:**
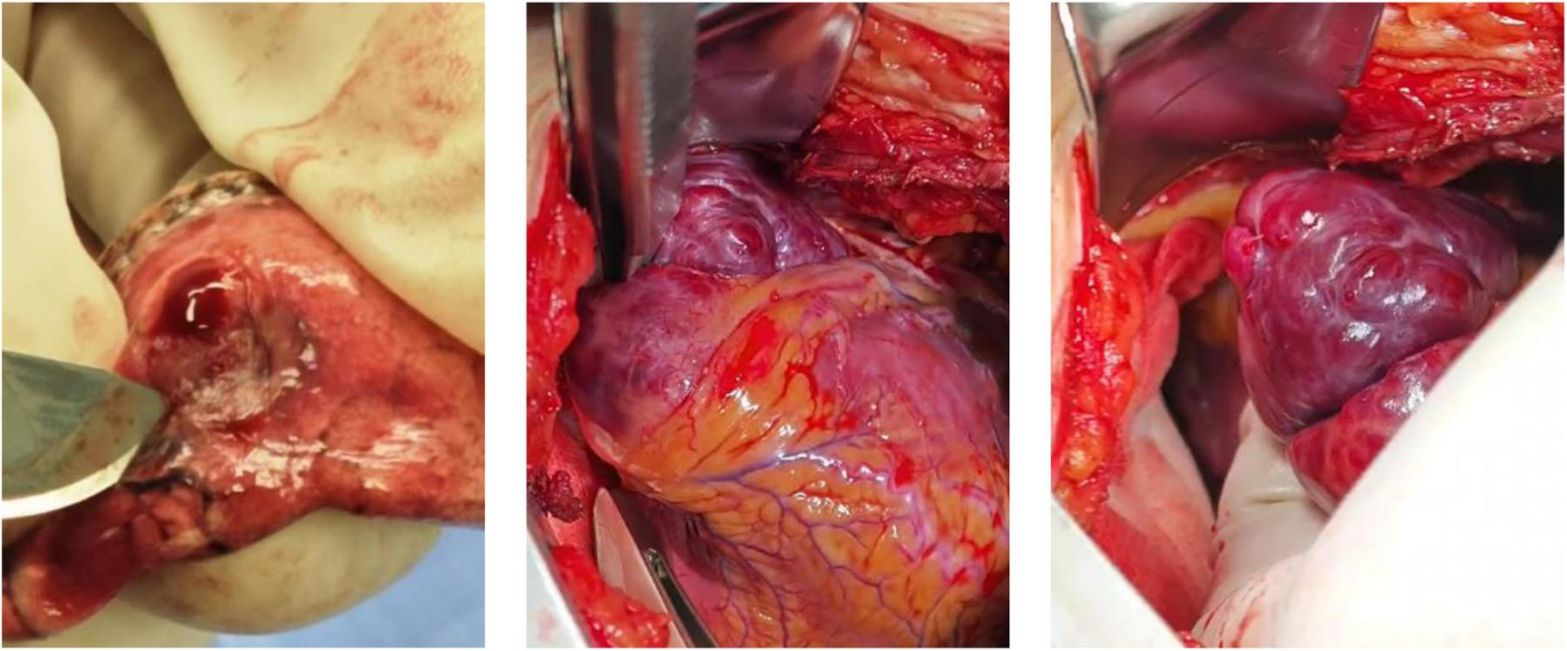
The intraoperative exploration situation.

Note: Given that the morphology of the ventricular mass resembled an angioma, and the rapid pathological findings of the two pulmonary nodules indicated vascular-like tissue hyperplasia, from the perspective of a unitary diagnosis, also supported the diagnosis of angioma for the ventricular mass, proceeding with a biopsy was considered to carry a high risk of uncontrollable bleeding. Additionally, considering the imaging finding that the left anterior descending coronary artery traversed the ventricular mass, a biopsy carried a risk of coronary artery injury, potentially leading to myocardial ischemia or infarction. Therefore, intraoperative biopsy of the ventricular mass was not performed (Figure 8).

## 4 Postoperative pathology and diagnosis

### 4.1 (Upper lobe) & (left lower lobe) wedge resection specimens

Microscopic examination reveals vascular-like tissue hyperplasia within the pulmonary parenchyma, accompanied by smooth muscle tissue hyperplasia in the surrounding areas. Based on hematoxylin and eosin (H&E) staining and immunohistochemistry, lymphangioleiomyomatosis is the primary consideration.

### 4.2 Immunohistochemistry

**Figure 9:**
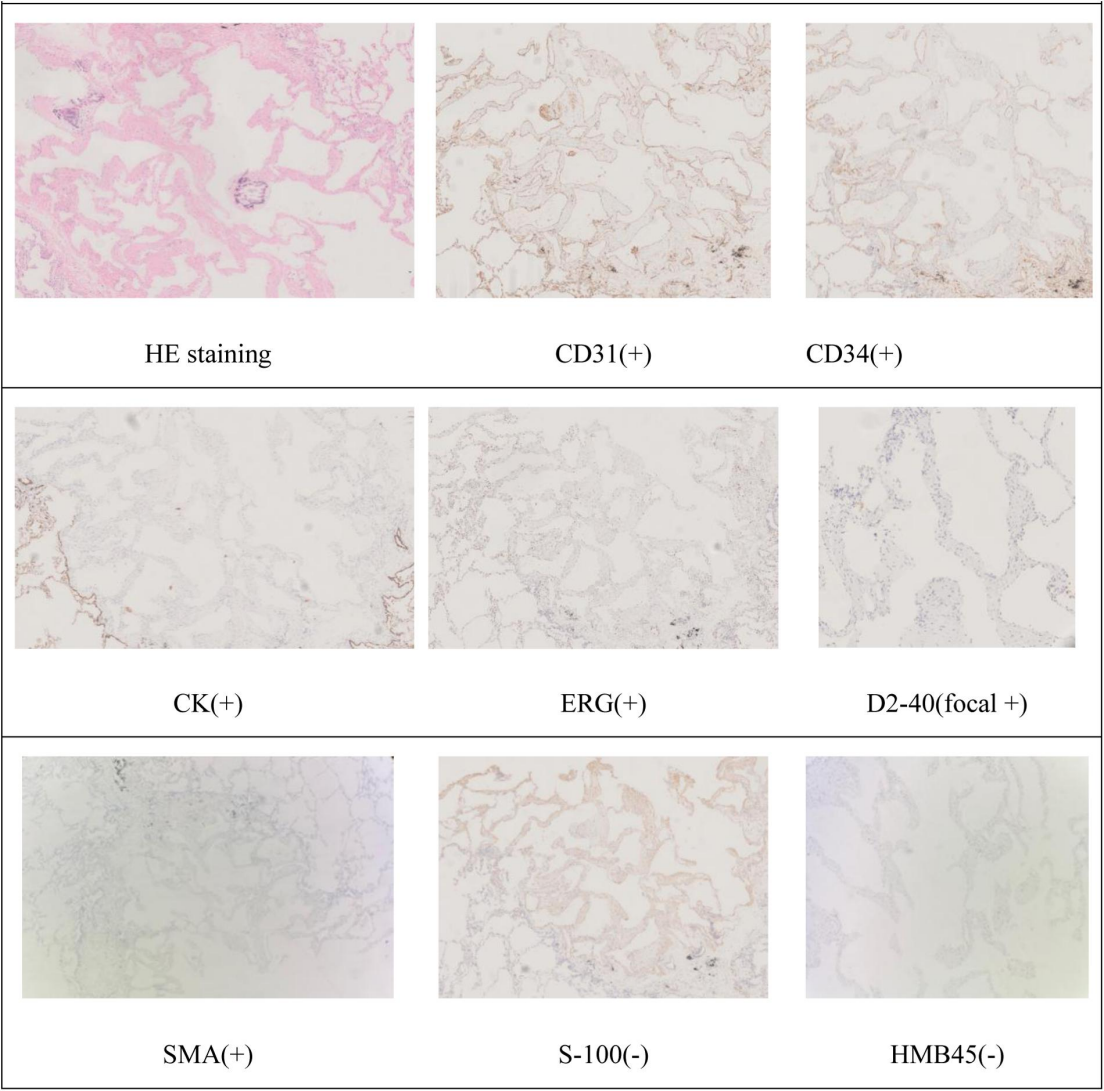
Pathology.

CD31(+), CD34(+), CK(+), D2-40 (focal +), Desmin(+), ERG(+), FLI-1(+), SMA(+), S-100(-), TTF-1(-), HMB45(-) (Figure 9).

## 5 Postoperative follow-up

The patient has now been followed for over one year postoperatively. Follow-up examinations showed no significant changes in the size, density, or morphology of the remaining pulmonary nodules or the paraventricular mass. Additionally, there were no signs of new nodules or tumor development in any other organs of the body.

## 6 Discussion

This study reports a rare case of angioma simultaneously involving the cardiac region and both lungs. The patient was an asymptomatic woman of reproductive age with incidental imaging findings. The diagnostic and therapeutic process highlights the complexity of differential diagnosis for space-occupying lesions in multiple organs and high-risk locations, as well as the central importance of interdisciplinary collaboration and individualized strategies.

### 6.1 Establishing the diagnosis: integration of clinical, imaging, and pathological evidence

The final diagnosis in this case relied on a closed-loop integration of clinical assessment, imaging characteristics, intraoperative findings, and definitive pathological evidence. Subtlety of Clinical Presentation: The patient had no respiratory or cardiovascular symptoms, consistent with the clinical features of most benign angiomas. However, this also increased the difficulty of differentiating it from other indolent diseases.

Guidance and Limitations of Imaging: CT and MRI clearly demonstrated multiple lesions in the cardiac region and both lungs. Coronary CTA revealed that the left anterior descending artery traversed the cardiac mass, a critical finding that directly informed the decision to avoid percutaneous biopsy. The PET/CT examination revealed an increase in glucose metabolism at the lesion site, suggesting that its nature might be benign. However, due to the physiological uptake of 18F-fluorodeoxyglucose by the ventricles, the assessment of cardiac masses by PET/CT is challenged. This physiological myocardial uptake can be effectively reduced by a combination of prolonged fasting, ketogenic diet preparation, and preoperative administration of unfractionated heparin before PET imaging (4). Adopting these standardized protocols may help minimize myocardial 18FDG accumulation and improve the diagnostic accuracy of cardiac mass assessment in future clinical practice.

The Gold Standard Role of Pathology: The histopathological and immunohistochemical results from the thoracoscopic wedge resection of the pulmonary nodules served as the cornerstone of the diagnosis. Microscopic examination revealed vascular-like tissue hyperplasia accompanied by smooth muscle hyperplasia in the surrounding area. Immunohistochemistry showed positivity for CD31 and CD34, confirming vascular endothelial origin; positivity for SMA and Desmin suggested the presence of smooth muscle components in the proliferating vessel walls; negativity for HMB45 effectively ruled out tumors with pericytic differentiation (such as hemangiopericytoma) and tumors with melanocytic differentiation (such as certain subtypes of angiomyolipoma or lymphangioleiomyomatosis). This immunophenotype (CD31+/CD34+/SMA+/HMB45-) is a typical feature supporting the diagnosis of angioma.

### 6.2 Analysis of key differential diagnoses

Differentiation from Lymphangioleiomyomatosis (LAM): LAM is a primary differential diagnosis in women of reproductive age. Its pulmonary manifestations are typically diffuse thin-walled cysts, but can also present as nodules. The key differentiating point lies in immunohistochemistry: LAM characteristically expresses the melanocytic marker HMB45 (5, 6), whereas it was negative in this case. Furthermore, the smooth muscle proliferating cells associated with LAM usually exhibit unique morphology, and D2-40 is diffusely positive (indicating lymphatic differentiation). In this case, D2-40 was only focally positive, which is more consistent with incidental lymphatic differentiation or admixture in angioma.

Differentiation from Pulmonary Epithelioid Hemangioendothelioma (PEH): PEH can also present as multiple bilateral pulmonary nodules. Its tumor cells are epithelioid and express vascular markers such as CD31 and CD34 on immunohistochemistry, but they typically do not express smooth muscle markers (e.g., SMA, Desmin) (7, 8). The clear positivity for SMA and Desmin in this case strongly supports the diagnosis of angioma with significant smooth muscle components in the vessel walls, rather than PEH.

Differentiation from Other Vascular Tumors: For example, angiosarcoma is malignant, usually grows rapidly, and exhibits aggressive imaging features, which are inconsistent with the long-term stable, benign course in this case. Hemangiopericytoma lacks the morphology of smooth muscle hyperplasia, typically expresses melanocytic markers (HMB45, MelanA), and does not express cytokeratin (CK) or CD10 (9–11), differing from the immunophenotype observed in this case.

Differentiation from Metastatic Tumors: The patient's tumor markers were all within normal limits, PET/CT showed no other primary tumor with increased metabolism, and the pathological morphology was entirely inconsistent with metastatic carcinoma or sarcoma, thus essentially ruling out metastasis.

### 6.3 Individualized treatment strategy and the application of the unitary principle

The treatment decision in this case reflects a highly individualized and risk-averse approach. Due to the proximity of the cardiac mass to the coronary artery, percutaneous or excisional biopsy carries an extremely high risk, potentially life-threatening. Therefore, the treatment team adopted a strategy of “starting from the easier and proceeding outward to inward”: first, safely obtaining lung tissue via thoracoscopy to clarify the pathological nature. Based on the clinical reasoning of the unitary principle, given the simultaneous imaging appearance of both the pulmonary nodules and the cardiac mass, and their shared benign vascular characteristics, it was highly inferred that the cardiac mass was a homologous angioma. Considering that angiomas are typically benign and slow-growing, and after obtaining definitive pathology (from the lung) and excluding malignancy, opting for close monitoring of the high-risk cardiac lesion rather than aggressive intervention was a reasonable decision aligned with the patient's best interests. Although watchful waiting and close surveillance may be sufficient for asymptomatic, benign lesions such as in the present case, some patients may present with masses that interfere with cardiac function or exhibit more aggressive behavior. In such instances, medical therapy, for example with propranolol, may be considered as a valuable therapeutic option (12). The stable one-year postoperative follow-up has further confirmed the appropriateness and safety of the conservative strategy adopted in this patient.

### 6.4 Implications and limitations of this case

This case provides the first systematic description of hemangioma presenting with the unique pattern of “cardiac mass accompanied by multiple bilateral pulmonary nodules,” thereby expanding the known clinical spectrum of hemangioma as a multisystemic disease. It strongly suggests that in clinical practice, when encountering multiple cardiopulmonary masses, hemangioma should be included in the routine differential diagnosis list.

The primary limitation of this study lies in the lack of direct histopathological confirmation of the cardiac lesion. However, under the strict adherence to medical ethics and the prioritization of patient safety, the comprehensive clinical diagnosis made based on a robust chain of indirect evidence, including characteristic imaging correlations, the gold-standard pathological diagnosis of the pulmonary lesions, and the benign natural course observed during follow-up, carries high credibility and significant clinical reference value. This approach offers a valuable reference for managing similar lesions in high-risk anatomical locations.

## 7 Conclusion

In summary, this case report details and analyzes a rare instance of multicentric angioma involving both the heart and lungs. The diagnosis was established through close collaboration within a multidisciplinary team, the safe implementation of minimally invasive thoracoscopic surgery, and, most critically, comprehensive immunohistochemical analysis. This case underscores the pivotal role of the immunophenotype (CD31+, CD34+, SMA+, and HMB45-) in differentiating vascular tumors, particularly in distinguishing angioma from conditions such as LAM and PEH. For high-risk masses involving critical anatomical structures, an individualized treatment strategy that prioritizes patient safety is paramount. This report provides valuable insights and experience for the clinical management of similarly complex cases.

## Data Availability

The original contributions presented in the study are included in the article/supplementary material, further inquiries can be directed to the corresponding author.
